# Supplementary material for: A registration strategy to characterize DTI-observed changes in skeletal muscle architecture due to passive shortening
Source: bioRxiv. 2024 Apr 14:2024.04.11.589123. Preprint. [Version 1] doi: 10.1101/2024.04.11.589123 (PMC11030449; doi:10.1101/2024.04.11.589123)
Supplement: Supplement 1 [file NIHPP2024.04.11.589123v1-supplement-1.pdf]

bioRxiv preprint doi: https://doi.org/10.1101/2024.04.11.589123; this version posted April 14, 2024. The copyright holder for this preprint (which was not certified by peer review) is the author/funder, who has granted bioRxiv a license to display the preprint in perpetuity. It is made available under aCC-BY 4.0 International license.

Supplemental Figures

Supplemental Figure legend 1.

Bland-Altman Plots showing the similarity between the original and transformed fiber-tracts for pennation angle ($\Delta\theta$) and fiber-tract length (ΔL_{FT}), on a fiber tract level, for high similarity (left) and low similarity (right) dataset. Note differences in Y-axis scales between the left and right panels.

Supplemental figure 2.

Dot plots displaying the correlation between the registration quality measures and the difference in pennation angle ($\Delta\theta$) between the original and transformed fiber-tracts for each of the participants (black dots).

Supplemental figure 3.

Dot plots displaying the correlation between registration quality measures and the difference in fiber-tract length (ΔL_{FT}) between the original and transformed fiber-tracts for each of the participants (black dots).

Supplemental Figure legend 4.

Scatterplots showing the relation between the similarity (S_i) and the difference in pennation angle ($\Delta\theta$) and fiber-tract length (ΔL_{FT}) between the original and transformed fiber-tracts for each of the individual fiber-tracts for the dataset with the highest and lowest averaged similarity value.

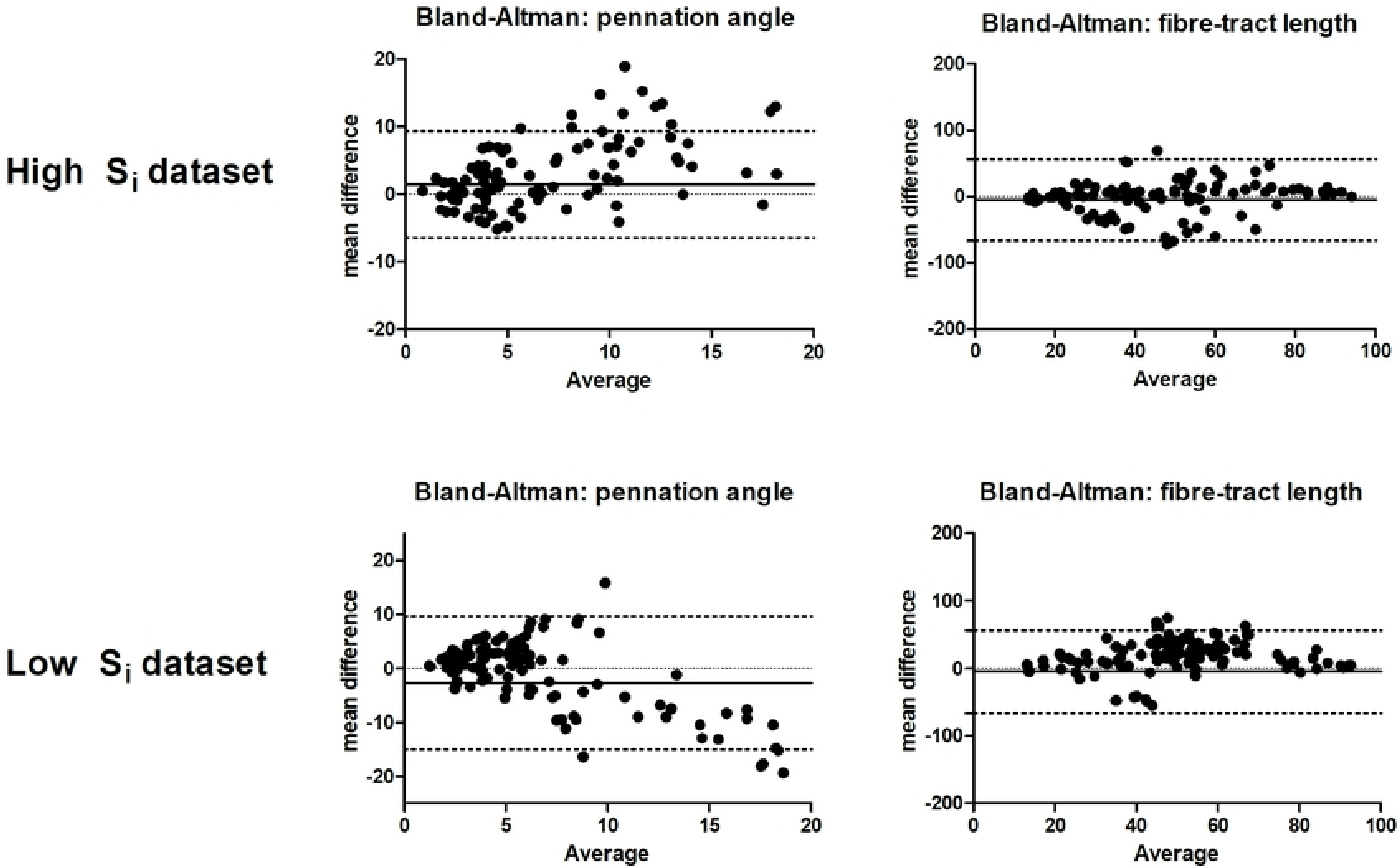

Supplemental Figure 1

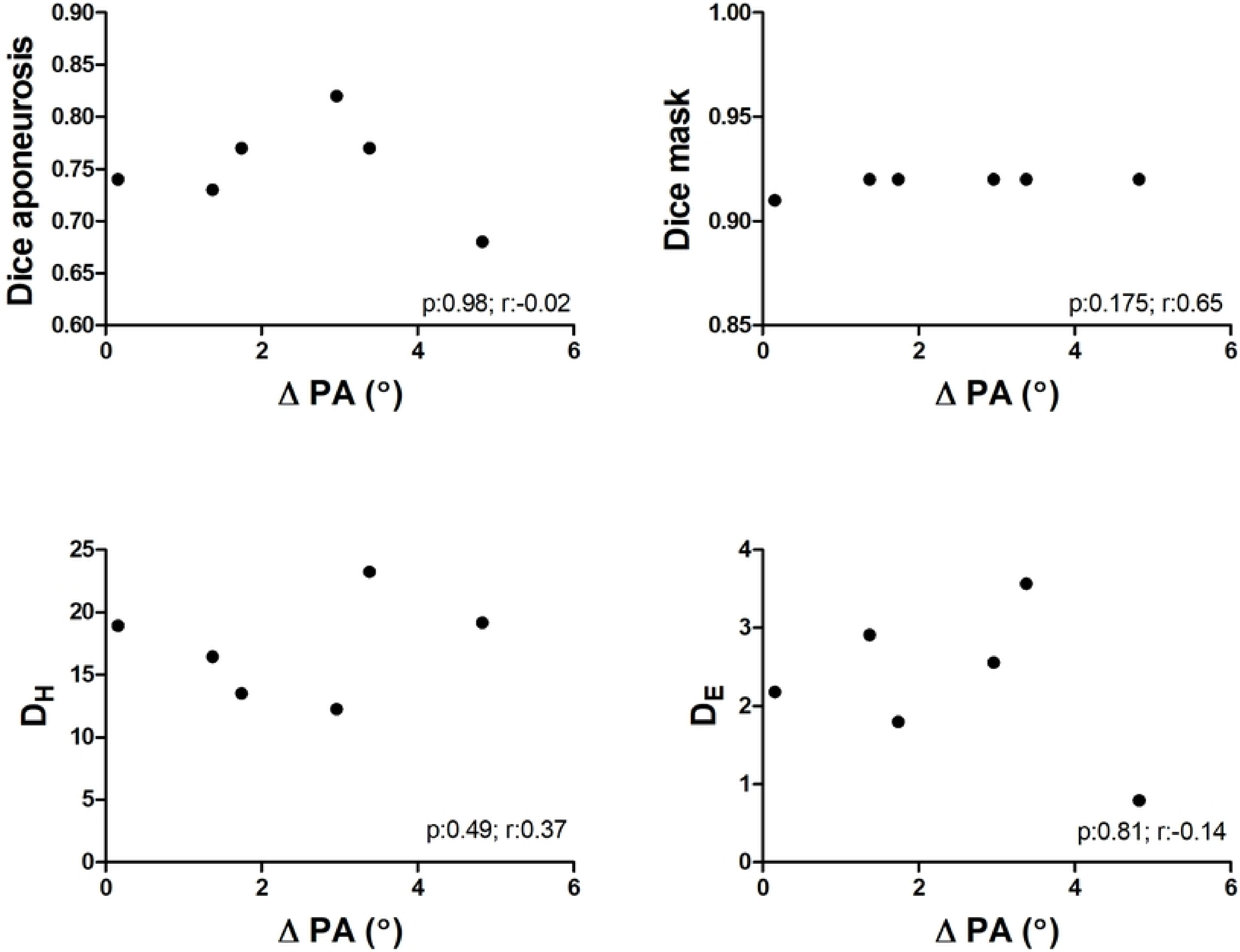

Supplemental Figure 2

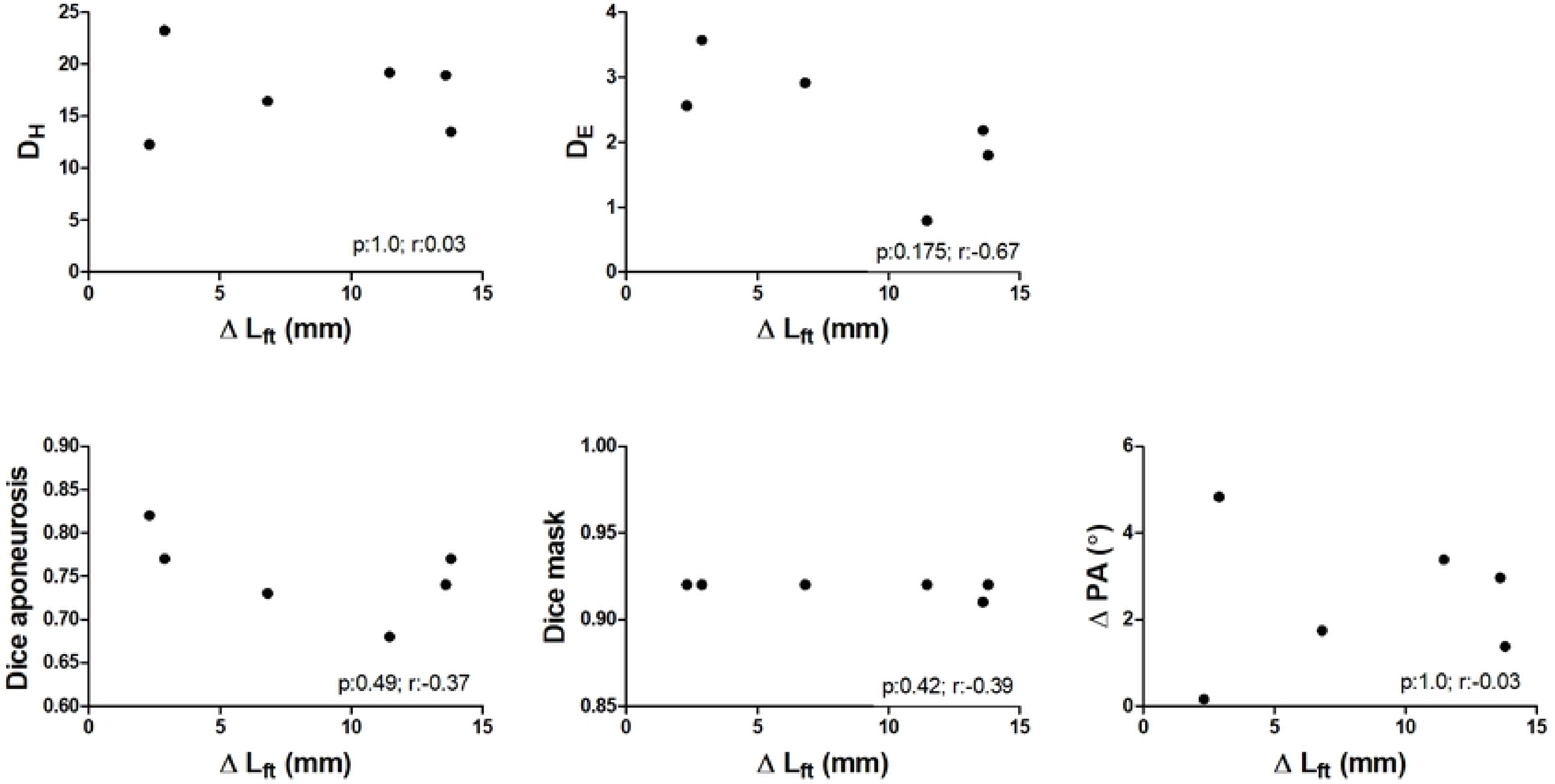

Supplemental Figure 3

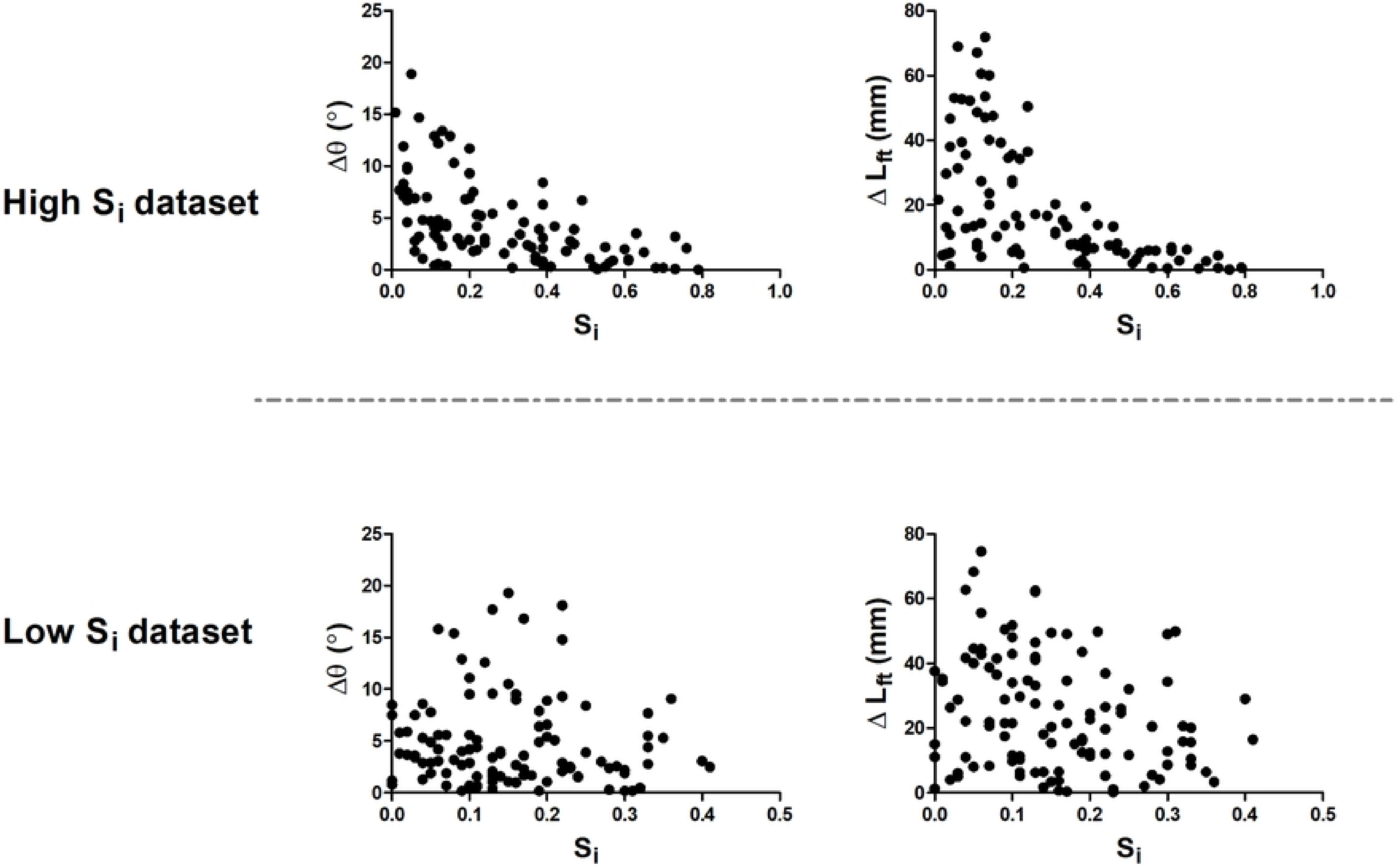

Supplemental Figure 4
